# Seafood and Water Management

**DOI:** 10.3390/foods3040622

**Published:** 2014-12-05

**Authors:** Saskia M. van Ruth, Erwin Brouwer, Alex Koot, Michiel Wijtten

**Affiliations:** 1 RIKILT Wageningen UR, P.O. Box 230, 6700 EV Wageningen, The Netherlands; E-Mails: erwin.brouwer@wur.nl (E.B.); alex.koot@wur.nl (A.K.); michiel.wijtten@wur.nl (M.W.); 2 Food Quality and Design Group, Wageningen University, P.O. Box 17, 6700 AA Wageningen, The Netherlands

**Keywords:** cod, fish, crude protein, moisture, pangasius, salmon, shrimp, tilapia, water

## Abstract

Seafood is an important food source for many. Consumers should be entitled to an informed choice, and there is growing concern about correct composition labeling of seafood. Due to its high price, it has been shown to be vulnerable to adulteration. In the present study, we focus on moisture levels in seafood. Moisture and crude protein contents of chilled and frozen cod, pangasius, salmon, shrimp and tilapia purchased from various retail outlets in the Netherlands were examined by reference methods and the values of which were compared with the reported data from other studies in literature. Significant differences in proximate composition were determined for different species and between chilled and frozen products of the same species. Pangasius products showed the highest moisture contents in general (86.3 g/100 g), and shrimp products revealed the largest differences between chilled and frozen products. Comparison with literature values and good manufacturing practice (GMP) standards exposed that, generally, chilled pangasius, frozen pangasius and frozen shrimp products presented considerably higher moisture and lower crude protein/nitrogen contents than those found in other studies. From the GMP standards, extraneous water was estimated on average at 26 g/100 g chilled pangasius product, and 25 and 34 g/100 g product for frozen shrimp and pangasius products, respectively.

## 1. Introduction

Seafood is an important source of nutrients in the human diet in various places in the world and comprises valuable proteins and lipids [1]. European food law underpins the concept of informed consumer choice in the purchase of food (Regulation (EC) No. 178/2002; [2]). The European Food Labelling Directive includes the requirement for a Quantitative Ingredients Declaration, which means that most pre-packed fish products are required to be labelled with a declaration of the amount of fish present as the percentage of the final weight of the product. There is, however, growing concern regarding the correct composition and labeling of seafood. Due to its premium price, seafood is susceptible to mislabeling, e.g., 75 fraud cases have been reported in the U.S. Pharmacopeial Convention food fraud database (http://www.foodfraud.org) to date [3]. Most often, this concerns replacement of species, replacement of wild products by farmed products, adulterated geographical origin and excessive water addition. In the European Rapid Alert System for Food and Feed [4], fish and fish products are listed 86 times in conjunction with fraud/adulteration. This system concerns mostly fraudulent health certificates and illegal imports. Food adulteration appears when a number of criteria are fulfilled. This requires an opportunity (a suitable target), motivation (economic, social and moral drivers), rationalization (justification by those involved) and lack of guardianship. In the very nature of food fraud, the actor conspires to manipulate product composition in the attempt to evade quality assurance and quality control plans implemented by manufacturers, distributors and purchasers and, therefore, requires a different approach from food safety assurance [3]. Food supply networks are optimized for rapid, low-cost production from all sources, which has consequently resulted in nontransparent, fragile systems with little guardianship when it comes to fraud. This certainly holds for seafood, which is sourced from many different parts around the globe and is often supplied through extensive intersecting networks.

Seafood proximate chemical composition is commonly categorized in water, protein, lipid and ash. Low levels of carbohydrates may be present, but the concentrations are considered negligible. The body composition varies and is associated with the fish appetite, growth and efficiency of feed utilization [5]. Water is the main component, both in volume and weight, in all seafood products. It is a determinant of the value of the products, sensory attributes and shelf-life. Commercial practices have evolved to retain and add moisture to seafood during harvest, processing and storage in order to reduce moisture or drip loss during frozen storage and thawing. However, there is a thin line between water addition to make up for moisture losses and excessive extraneous water addition for one’s own economic gain. Water and water binder addition are technologies borrowed from the meat industry.

The second major seafood component is protein. The protein content of foods is commonly derived by calculation, based on the measurement of nitrogen content using the Kjeldahl method [6]. The nitrogen in fish is distributed between proteins and other nitrogen-containing compounds, which is often referred to as non-protein nitrogen (NPN). The hundreds of proteins in muscle consist of the contractile proteins,* i.e.*, the myofibrillar proteins; the sarcoplasmic proteins from the fluid within the muscle cells are mainly enzymes and are the only water-soluble proteins; and the stromal proteins, which are present in the connective tissue and hold the muscle bundles together and to the skeleton, and include proteins that are associated with cellular membranes. NPN compounds are comprised of peptides, amino acids, amines, amine oxides, guanidine compounds, quaternary ammonium compounds, purines and urea. These compounds originate mostly from the sarcoplasm. Nitrogen levels vary with the different fish species, but most finfish muscle tissue consists of 18–22 g crude protein per 100 g product; converted, this is approximately 2.9–3.5 g nitrogen per 100 g product [7].

For physiological reasons, strong relationships exist between protein and moisture levels in meat [8] and seafood [5,9]. In general, in muscle meat, the water content is close to 77% and protein to 23%, which results in a water-to-protein ratio of 3.35. This information is also used to control excessive water addition. The Poultry Meat Marketing Standards Regulation (EC) 543/2008 [10] regulates the amounts of extraneous water in poultry meat and limits the water-to-protein ratio to 3.40 for chicken breasts (non-preparations). Furthermore, the Food Industries Manual states limits for water-to-protein ratios on a fat-free basis for pork (3.40–3.50) and beef (3.60–3.70) [11]. In addition to the relationship between moisture and protein contents, correlations with lipid content may exist, as well.

Considering the vulnerability of seafood to adulteration and the simplicity of the measurements, surprisingly few reports are available surveying the actual moisture/protein contents of seafood products on the market. Therefore, the current study aims for insights into common water management practice for a variety of seafood species on the Dutch market, representing a considerable share of the seafood products consumed in the Netherlands. For this study, seafood species were selected that were reported to be among those most frequently consumed in the Netherlands in 2013 [12] and are sold chilled and frozen,* i.e.*, cod, pangasius, salmon, shrimp and tilapia products. The moisture and crude protein contents of the products obtained from supermarkets, specialty shops and open markets (mobile outlets) were examined with the official reference methods and evaluated against values in the literature.

## 2. Experimental Section

### 2.1. Sample Material

Seafood samples (110) of five species, sold as chilled and frozen (55/55), were purchased at various retail outlets (supermarkets, specialty seafood shops, open air markets) in the Netherlands in spring, 2014 (Table 1). On arrival to the laboratory, all samples were labeled and stored at −18 °C. Samples were ground prior to moisture and crude protein analysis.

**Table 1:** Sampling design.

Variable	Categories	Number of Samples
Seafood species	Cod (*Gadus morhua*)	20
	Pangasius (*Pangasius bocourti*)	20
	Salmon (*Salmo salar*)	26
	Shrimp (*Crangon* spp., *Penaeus* spp., *Pandalus* spp., peeled)	24
	Tilapia (*Oreochromis* spp.)	20
Sales temperature	Chilled	55
	Frozen	55
Retail outlet	Supermarket, convenience	26
	Supermarket, intermediate	42
	Supermarket, discounter	12
	Specialty shop	18
	Open air market	12

### 2.2. Moisture Analysis

Moisture contents were determined by the reference method, ISO 1442:1997 [13]. This method considers the loss in mass obtained after thorough mixing of the test portion with sand and drying to constant mass at 103 ± 2 °C, divided by the mass of the test portion. Moisture analyses were carried out on each individual sample in duplicate.

### 2.3. Crude Protein Analysis

Crude protein contents were determined by the reference method, ISO 937:1978 [14]. This Kjeldahl method involves the digesting of a test portion with concentrated sulfuric acid, using copper (II) sulfate as a catalyst, to convert organic nitrogen to ammonia ions. Then, alkalization, distillation of the liberated ammonia into an excess of boric acid solution and titration with hydrochloric acid to determine the ammonia bound by the boric acid are followed by the calculation of the nitrogen content of the sample from the amount of ammonia produced. A standard factor, termed the nitrogen factor, of 6.25 was and is typically used in the determination of crude protein in foods, based on the assumption that the average nitrogen content of proteins is 16% (1/0.16 = 6.25). Protein analyses were carried out on each individual sample in duplicate. Protein values reported in the publication refer to these crude protein analyses.

### 2.4. Statistical Analysis

Moisture and protein content data were subjected to multi-factor analysis of variance (MANOVA; factors: species and sales temperature), and Fisher’s least significant difference (LSD) tests were carried out to determine significant differences among groups using XLSTAT 2014.3.02 (Addinsoft, Paris, France). MANOVA can assess two or more independent variables (in this case, species and sales temperature) for the significance of the effects on two or more metric dependents (in this case, moisture and protein contents). This allows a joint analysis of each dependent rather than performing several univariate tests, thus avoiding multiple testing risks. A significance level of *p <* 0.05 was used throughout the study.

## 3. Results and Discussion

### 3.1. Water and Protein Contents

**Figure 1:**
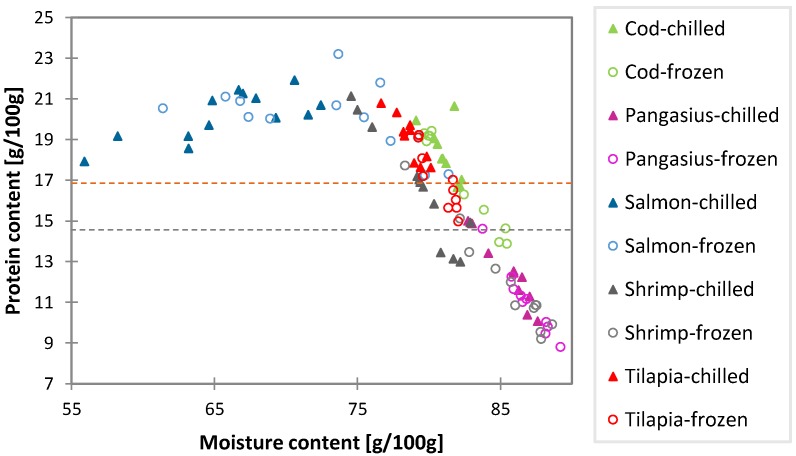
Moisture* versus* protein contents for individual samples of five seafood species (different colors), for both chilled (filled triangle) and frozen products (open circle). Horizontal lines indicate protein levels associated with minimal nitrogen factors for GMP (good manufacturing practice) fish ingredients for white fish (orange) and washed and peeled shrimp (grey) [7].

The moisture and protein levels of five different seafood species, sold chilled and frozen, from various retail outlets were analyzed with the ISO reference methods. In the current study, the moisture content assessment was based on oven drying, but also, other technologies are generally available for moisture determinations, e.g., near-infrared, nuclear magnetic resonance and guided microwave spectroscopy [15,16,17]. The protein (nitrogen) levels were determined by the widely-used Kjeldahl methodology. Alternatives for determining the nitrogen content are the Dumas combustion method, as well as colorimetric, electrophoretic, chromatography, mass spectrometry and immunology-based methods [18]. The moisture and protein contents of the seafood samples analyzed in the current study are presented for the individual 110 samples in Figure 1.

Mean values and statistical comparison of the species and chilled/frozen samples are presented in Table 2. MANOVA indicated significant differences in moisture contents between the groups (*F* (5,109)* =* 70.7, *p <* 0.0001), to which both the factor, fish species (*F* (4,109) = 80.0, *p <* 0.0001), and the factor, sales temperature (*F* (1,109) = 33.7, *p <* 0.0001), contributed. Similarly MANOVA pointed out significant differences in protein contents between the groups (*F* (5,109) = 53.6, *p <* 0.0001), with also the factor, fish species (*F* (4,109) = 60.9, *p <* 0.0001) and the factor, sales temperature (*F* (1,109) = 24.4, *p <* 0.0001), having a significant effect on the protein contents.

Salmon samples showed the lowest moisture contents, which is due to the relatively high lipid fraction in comparison to the other samples,* i.e.*, white fish and shrimps. On the other hand, the protein contents of the salmon samples were relatively high, and the moisture contents were more variable. For tilapia and cod samples, significantly higher moisture and lower protein contents were observed compared to the salmon samples, and in turn, pangasius demonstrated significantly higher moisture and lower protein contents than tilapia and cod (Fisher’s LSD test, *p <* 0.05). Shrimp moisture and protein contents overlapped with those of the tilapia/cod and pangasius groups.

**Table 2:** Moisture contents, protein contents and water/protein ratios in various seafood samples presented for different groups (mean ± SD) ^1^.

Sample	Moisture Content (g/100 g)	Protein Content (g/100 g)	Water/Protein Ratio
All	79.4 ± 7.2	16.5 ± 3.7	4.8
Cod	81.6 ± 1.9 ^B,C^	17.6 ± 2.0 ^II^	4.6
Pangasius	86.3 ± 1.7 ^A^	11.7 ± 1.8 ^IV^	7.4
Salmon	69.3 ± 6.6 ^D^	20.1 ± 1.4 ^I^	3.4
Shrimp	82.4 ± 4.3 ^B^	14.2 ± 3.5 ^III^	5.8
Tilapia	79.7 ±1.6 ^C^	18.0 ±1.7 ^II^	4.4
Chilled	77.5 ± 7.5 ^Y^	17.4 ± 3.2 ^S^	4.4
Frozen	81.3 ± 6.2 ^X^	15.5 ± 4.0 ^T^	5.2

^1^ A–D, I–IV, X and Y, S and T, values with either different letters or different Roman numbers in a column are significantly different (MANOVA, Fisher’s LSD test *p <* 0.05).

The determined moisture and protein contents in the cod samples are in agreement with those reported by Krzynowek and Murphy [19], who reported a moisture content of 82.1% and a protein content of 17.4% for fresh fillets. Similarly, the salmon proximate composition is also in line with previous studies, e.g., a moisture content range of 60%–75% and a protein range of 17%–25% was reported in a study examining a large number of salmon samples [20]. Furthermore, a second large study analyzing 178 salmon samples from Ireland, Norway and Scotland (178 samples) resulted in an average moisture content of 69.1% moisture [17], which is all fairly close to the data gathered in the present study. The analyses of the pangasius, shrimp and tilapia samples revealed, on average, higher moisture and lower protein contents than reported in other studies. Rathod and Pagarkar [21] reported recently pangasius moisture and protein contents of 76.6% and 14.4%, respectively, and Karl and co-workers [22] similarly reported pangasius moisture and protein contents of 82.7% and 14.2%, respectively. For black tiger shrimp, moisture contents of 80.5% and protein contents of 17.1% have been reported, for white shrimp, 77.2% moisture and 18.8% protein [1], and for pink shrimp, moisture contents of 80.1% and protein contents of 18.1% [19]. Finally, for tilapia, moisture contents of 75.8% and protein contents of 18.8% [23], as well as a moisture range of 74.4%–77.8% have been published [24]. In conclusion, for the cod and salmon samples in the present study, moisture and protein contents are similar to those reported by other authors, whereas pangasius, shrimp and tilapia revealed, on average, higher moisture and lower protein contents.

The water and protein content of the chilled and frozen samples were statistically compared (Table 3). Both for moisture (*F* (9,109) = 46.3, *p <* 0.0001) and protein (*F* (9,109) = 36.8, *p <* 0.0001), significant differences between groups were observed (MANOVA). It is remarkable that for all species, frozen samples showed higher moisture and lower protein contents than the chilled samples. Salmon and shrimp sample groups presented significant differences in moisture content between chilled and frozen samples (Fisher’s LSD test, *p <* 0.05), and the shrimp and tilapia sample groups demonstrated also significant differences in protein content between chilled and frozen samples.

**Table 3:** Moisture contents, protein contents and water/protein ratios in various seafood samples (mean ± SD) ^1^.

Seafood Species	Sales Temperature	Moisture Content (g/100 g)	Protein Content (g/100 g)	Water/Protein Ratio
Cod	Chilled (*n* = 10)	81.1 ± 1.0 ^B,C^	18.3 ± 1.3 ^II,III^	4.4
	Frozen (*n* = 10)	82.2 ± 2.5 ^B^	17.0 ± 2.4 ^III,IV^	4.8
	Δ Frozen-chilled	+1.1 (+1.4%)	−1.3 (−7.1%)	+0.4 (+10.0%)
Pangasius	Chilled (*n* = 10)	85.6 ± 1.7 ^A^	12.4 ± 1.7 ^V^	6.9
	Frozen (*n* = 10)	86.9 ± 1.6 ^A^	11.0 ± 1.7 ^V^	7.9
	Δ Frozen-chilled	+1.3 (+1.5%)	−1.4 (−11.3%)	+1.0 (+14.4%)
Salmon	Chilled (*n* = 13)	65.8 ± 4.9 ^E^	20.2 ± 1.2 ^I^	3.3
	Frozen (*n* = 13)	72.9 ± 6.2 ^D^	20.1 ± 1.7 ^I^	3.6
	Δ Frozen-chilled	+7.1 (+10.8%)	−0.1 (−0.5%)	+0.3 (+10.0%)
Shrimp	Chilled (*n* = 12)	79.2 ± 2.7 ^C^	16.6 ± 2.8 ^IV^	4.8
	Frozen (*n* = 12)	85.5 ± 3.1 ^A^	11.9 ± 2.5 ^V^	7.2
	Δ Frozen-chilled	+6.3 (+8.0%)	−4.7 (−28.3%)	+2.4 (+50.0%)
Tilapia	Chilled (*n* = 10)	78.7 ±1.0 ^C^	19.0 ±1.1 ^I,II^	4.1
	Frozen (*n* = 10)	80.8 ± 1.2 ^B,C^	16.9 ± 1.5 ^III,IV^	4.6
	Δ Frozen-chilled	+2.1 (+2.7%)	−2.1 (−11.1%)	+0.5 (+12.0%)

^1^ A–D, I–IV, values with either different letters or different Roman numbers in a column are significantly different (MANOVA, Fisher’s LSD test *p <* 0.05).

Mean water-to-protein ratios varied from 3.3 to 4.4 for chilled cod, salmon and tilapia. Chilled shrimp (4.8) and pangasius (6.9) demonstrate relatively high mean water-to-protein ratios. Regardless of species, frozen samples had higher water-to-protein ratios than the chilled samples, with striking mean values for the shrimp and pangasius groups,* i.e.*, 7.2 and 7.9, respectively.

### 3.2. Comparison of Nitrogen Factors

In the U.K., the determination of nitrogen as a quantitative marker for seafood fat-free protein is well established and is the official chemical enforcement method. It is also widely used by food producers to check the specification and added water of their seafood raw materials. A “nitrogen factor” is the average nitrogen content of seafood tissues, on a fat-free basis, unless the fat content is low, as in white fish [25]. In the U.K., Code of Practice on the declaration of fish content in fish products [7], a nitrogen factor of 2.65 has been set for white fish (reflecting a protein content of 16.5 g/100 g in the present study) and a limit of 2.33 for washed and peeled shrimp (reflecting a protein content of 14.6 in the present study). Both lines are presented in Figure 1 and show that a considerable number of samples have lower nitrogen (protein) contents than these nitrogen (protein) factors. Furthermore, other relevant nitrogen factors for good manufacturing practice (GMP) fish ingredients of various species have been published recently and concern (minced) cod (2.67), pangasius (2.66) and tilapia (2.88) [25]. Salmon values are not considered here, since its higher lipid content would need to be taken into account to make appropriate comparisons. Table 4 presents the number of cod, pangasius, shrimp and tilapia products analyzed, which shows higher or lower nitrogen content values than the nitrogen factors published. Those samples not meeting the nitrogen factors were subjected to further estimation of the extraneous water based on these nitrogen factors: [(nitrogen factor − nitrogen content measured)/nitrogen factor] × 100%.

**Table 4:** Comparison of the nitrogen contents of seafood samples with Kjeldahl nitrogen factors for GMP fish ingredients [7,25]: the number of samples with nitrogen contents below or over minimal nitrogen factors ^1^.

Fish Species	Chilled Products		Frozen Products	
	Below Minimal Nitrogen Factor	Exceeding Minimal Nitrogen Factor	Below Minimal Nitrogen Factor	Exceeding Minimal Nitrogen Factor
Cod	1	9	5	5
Pangasius	10	0	10	0
Shrimp	3	9	10	2
Tilapia	3	7	7	3
Sum	17 (40%)	25 (60%)	32 (76%)	10 (24%)

^1^ The nitrogen factors considered are: cod (2.67 [25]), pangasius (2.66 [25]), shrimp (2.33 [7]) and tilapia (2.88 [25]).

Chilled cod and tilapia included some samples with nitrogen contents below the factors published, but only a marginal difference existed (0.2%–3%). Estimation of water replacement based on the nitrogen factors indicated that the chilled shrimp samples that did not meet the nitrogen factors comprised 9 g extraneous water/100 g and chilled pangasius 26 g extraneous water/100 g product. Only a single chilled product had water addition labelled. Water replacement in the frozen products with lower nitrogen contents than the nitrogen factors was estimated as well, and amounted to tilapia 10 g extraneous water/100 g product, for cod 14 g/100 g product, for shrimp 25 g/100 g product and for pangasius 34 g/100 g product, on average. Most of the frozen products not meeting the nitrogen factors had water addition labelled. The quantity indicated was usually 10% extraneous water (drip loss, including glazing), except for some shrimp products, which indicated 10%–20% added water. Nonetheless, the estimations indicated higher extraneous water levels, which implies under-labelling of the water addition, especially for frozen shrimp and pangasius products.

## 4. Conclusions

The present study in which 110 samples of seafood were analyzed for their moisture and protein contents showed significant differences in proximate composition for different species and products sales temperature (chilled or frozen). Chilled pangasius, frozen pangasius and frozen shrimp products revealed consistently higher moisture and lower protein/nitrogen contents compared to other studies in the literature, as well as compared to GMP standards. These results indicate water addition, which seems to occur unlabeled in certain chilled products and under-labelled in particular frozen products.
